# Prevalence and Blood Biochemical Parameters Associated with Sarcopenia Among Korean Postmenopausal Women Aged 50 Years and Older: A Nationwide Cross-Sectional Study

**DOI:** 10.3390/healthcare14203419

**Published:** 2026-10-13

**Authors:** Seongheui Kim

**Affiliations:** Department of Nursing, Daejin University, Pocheon 11159, Republic of Korea; nursingkim@daejin.ac.kr

**Keywords:** sarcopenia, postmenopausal women, KNHANES, AWGS 2025

## Abstract

**Highlights:**

**What are the main findings?**

• Using the AWGS 2025 age-specific criteria, the weighted prevalence of sarcopenia was 10.5% among Korean postmenopausal women aged ≥50 years and 9.7% among those aged 50–64 years.
• Higher triglycerides were associated with higher sarcopenia odds after adjustment for age, BMI, and age at menopause; higher hemoglobin was associated with lower odds after adjustment for age.

**What are the implications of the main findings?**

• The findings highlight the importance of attention to muscle health among postmenopausal women beginning in midlife.
• Blood biochemical parameters may have potential relevance to muscle health assessment among postmenopausal women, but their clinical utility requires further validation.

**Abstract:**

**Background/Objectives:** Sarcopenia is a growing health concern from midlife, but evidence on its biochemical correlates among postmenopausal women younger than 65 years remains limited. This study aimed to estimate sarcopenia prevalence and explore associations with blood biochemical parameters among Korean postmenopausal women aged ≥50 years using the Asian Working Group for Sarcopenia (AWGS) 2025 age-specific diagnostic criteria. **Methods:** We analyzed 2024 Korea National Health and Nutrition Examination Survey data from 1157 postmenopausal women aged ≥50 years with complete muscle and biochemical measurements. The 13 biochemical parameters were glycated hemoglobin, total cholesterol, high-density lipoprotein cholesterol, triglycerides, hemoglobin, creatinine, aspartate aminotransferase, high-sensitivity C-reactive protein, calcium, phosphorus, magnesium, parathyroid hormone, and serum 25-hydroxyvitamin D_3_. Complex sample logistic regression examined each parameter’s association with sarcopenia, adjusting for age alone or age, body mass index (BMI), and age at menopause. **Results:** The weighted prevalence of sarcopenia was 10.5% overall and 9.7% among women aged 50–64 years. Triglycerides were not significantly associated with sarcopenia in the age-adjusted model. However, in the model adjusted for age, BMI, and age at menopause, each 10 mg/dL increase in triglyceride levels was associated with approximately 5.1% higher odds of sarcopenia (aOR = 1.051, 95% CI: 1.020–1.072, *p* < 0.001). Higher hemoglobin levels were associated with lower odds of sarcopenia in the age-adjusted model (aOR = 0.734, 95% CI: 0.599–0.901, *p* = 0.003). Both associations remained significant after false discovery rate correction. **Conclusions:** These findings highlight the importance of muscle health before older age in postmenopausal women. Biochemical parameters may have potential relevance to muscle health assessment, but their clinical utility requires further validation.

## 1. Introduction

### 1.1. Rationale for the Study

Sarcopenia is characterized by reductions in muscle mass and strength [1] and is an important health condition associated with impaired physical function and increased risks of falls, fractures, disability, metabolic disorders, and mortality [1,2,3]. Although the risk of sarcopenia increases with age, growing attention has been directed toward muscle health in midlife, extending the focus beyond older adults [4]. The prevalence of sarcopenia has been reported to range from 10% to 27%, depending on the diagnostic criteria and cutoff values applied [5]. Among adults younger than 60 years, prevalence estimates ranged from 8% to 36% across definitions, including those based on muscle mass alone [5]. These findings suggest that attention to sarcopenia prevention may be warranted before older age.

Postmenopausal women experience menopause-related hormonal and body-composition changes in addition to age-related alterations in skeletal muscle [6]. Estrogen contributes to the regulation of muscle protein metabolism, regeneration, and mitochondrial function [7]. The menopausal transition has been associated with increased fat mass and decreased lean mass [6]. A systematic review and meta-analysis found that early menopause was associated with lower muscle mass, whereas premature ovarian insufficiency was associated with lower handgrip strength and poorer physical performance [8]. These findings suggest that muscle health in postmenopausal women should be considered in relation to both aging and menopause-related changes, highlighting the importance of assessing sarcopenia and its associated factors from midlife onward.

This perspective is also reflected in the recently revised Asian Working Group for Sarcopenia (AWGS) 2025 criteria. AWGS 2025 revised the existing diagnostic framework, which considers the characteristics of Asian populations, and expanded sarcopenia assessment beyond older adults to include middle-aged adults aged 50–64 years [9]. It emphasized a life-course approach to assessing and managing muscle health before substantial functional decline develops in older age [9]. In a Korean study applying the AWGS 2025 criteria, the prevalence of low muscle mass among women was 3.2% at ages 50–54 years, 3.4% at ages 55–59 years, and 3.3% at ages 60–64 years, indicating that low muscle mass was already present among women in their 50s, with higher prevalence in older age groups, reaching 35.4% among women aged ≥80 years [4].

Previous Korean studies have examined sarcopenia among older adults [10,11]. However, studies applying both muscle mass and muscle strength according to the AWGS 2025 criteria among postmenopausal women from midlife through older adulthood remain limited. A recent study applying the AWGS 2025 criteria to the same Korea National Health and Nutrition Examination Survey (KNHANES) dataset also focused on the prevalence of low muscle mass and patterns by age and sex among Korean adults [4].

Muscle health is associated with various physiological factors [1]. Blood glucose and lipid indicators reflect metabolic status that may be associated with muscle health [1,12]. Hemoglobin reflects oxygen-carrying capacity, and lower hemoglobin levels have been associated with sarcopenia or impaired muscle function [13,14]. An international systematic review and meta-analysis also found higher blood C-reactive protein levels in individuals with sarcopenia than in those without sarcopenia, providing a rationale for including inflammatory indicators [15]. The relationships between minerals and muscle health have also been investigated; however, the consistency and sufficiency of the evidence vary across indicators, warranting further investigation among postmenopausal women [16]. Significant associations between blood vitamin D levels and sarcopenia have been reported in both Korean and international studies, supporting the inclusion of vitamin D as a potential sarcopenia-related indicator [17,18]. Therefore, understanding the factors associated with sarcopenia requires examining a range of blood biochemical parameters reflecting metabolic, inflammatory, hematological, and nutritional and mineral status.

International diagnostic frameworks for sarcopenia include muscle strength in addition to muscle mass, and some also include physical performance [1]. In Asia, the AWGS criteria, which consider population characteristics, have been used. The recently revised AWGS 2025 consensus requires concurrent low muscle mass and low muscle strength for a diagnosis of sarcopenia and positions physical performance as an outcome measure [9]. In line with this international approach to muscle health assessment, this study applies the AWGS 2025 age-specific diagnostic criteria, extending assessment beyond older adults to include postmenopausal women aged 50–64 years. This approach is significant in that it assesses sarcopenia among postmenopausal women from midlife through older adulthood and examines its associations with blood biochemical parameters from multiple physiological domains measured in the same participants.

To this end, the study uses nationally representative data from the 2024 Korea National Health and Nutrition Examination Survey and accounts for the complex sampling design and survey weights in the analyses. Specifically, it uses objective examination data collected through standardized procedures, including biochemical blood test results, handgrip strength measurements, and appendicular skeletal muscle mass measured by dual-energy X-ray absorptiometry (DXA) or bioelectrical impedance analysis (BIA), to examine whether associations reported in previous Korean and international studies are also observed among Korean postmenopausal women, thereby contributing to an understanding of similarities and differences in these associations across populations and diagnostic criteria. The findings may provide foundational evidence for examining the potential use of biochemical parameters in muscle health assessment and preventive health management among postmenopausal women from midlife through older adulthood.

Accordingly, this study aims to estimate sarcopenia prevalence and explore associations between various blood biochemical parameters and sarcopenia among Korean postmenopausal women aged ≥50 years.

### 1.2. Study Objectives

The specific objectives of this study were as follows:

• To estimate the weighted prevalence of sarcopenia among Korean postmenopausal women aged ≥50 years, overall and by age group.
• To examine differences in general characteristics, including menopause-related characteristics, between postmenopausal women with and without sarcopenia.
• To examine differences in blood biochemical parameters between postmenopausal women with and without sarcopenia.
• To examine associations between blood biochemical parameters and sarcopenia in a model adjusted for age and a model adjusted for age, body mass index (BMI), and age at menopause.

## 2. Methods

### 2.1. Study Design and Data Source

This cross-sectional study used raw data from the third year of the ninth Korea National Health and Nutrition Examination Survey (KNHANES IX-3), conducted in 2024 by the Korea Disease Control and Prevention Agency [19]. The KNHANES is designed to produce nationally representative estimates of the health and nutritional status of the Korean population. It employs a two-stage stratified cluster sampling design, with enumeration districts and households serving as the first- and second-stage sampling units, respectively.

### 2.2. Study Participants

**Figure 1:**
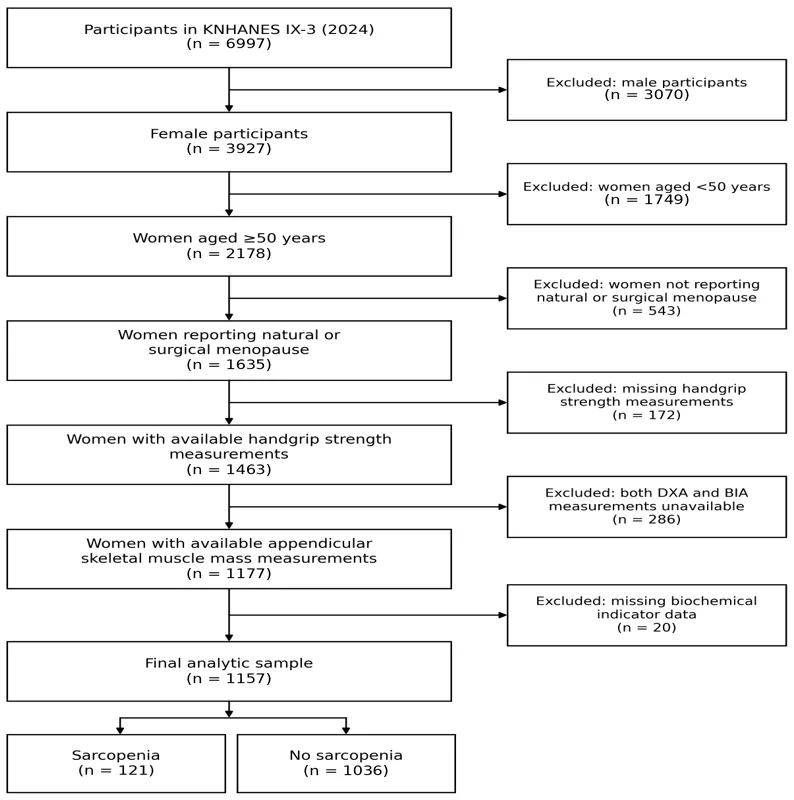
Flowchart of participant selection. In the final analytic sample, appendicular skeletal muscle mass was assessed using DXA in 1070 women and BIA in 87 women without DXA measurements. The survey-weighted estimated population was 6,330,964 Korean postmenopausal women aged ≥50 years.

This study included postmenopausal women aged ≥50 years at the time of the survey because the AWGS 2025 diagnostic criteria for sarcopenia provide age-specific thresholds for adults aged 50 years and older [9]. The participant selection process is presented in Figure 1.

Among the 6997 participants in the ninth Korea National Health and Nutrition Examination Survey, third year (KNHANES IX-3, 2024), 2178 were women aged ≥50 years. Of these, 1635 self-reported currently being in natural or surgical menopause. The surgical menopause category included hysterectomy or bilateral salpingo-oophorectomy. After excluding 172 women with missing handgrip strength measurements required for sarcopenia diagnosis, 1463 women remained. An additional 286 women were excluded because neither DXA nor BIA measurements were available to determine appendicular skeletal muscle mass, leaving 1177 women. Of these, 20 women with missing blood biochemical parameter data were excluded, resulting in a final analytic sample of 1157 women. Among them, 121 had sarcopenia and 1036 did not. After applying complex survey weights, the estimated population comprised 6,330,964 Korean postmenopausal women aged ≥50 years.

DXA measurements of appendicular skeletal muscle mass were used preferentially to determine sarcopenia status, with BIA measurements used when DXA measurements were unavailable. Overall, DXA was used for 1070 participants (92.5%) and BIA for 87 participants (7.5%). Among the 556 participants aged 50–64 years, DXA was used for 527 (94.8%) and BIA for 29 (5.2%). Among the 601 participants aged ≥65 years, DXA was used for 543 (90.3%) and BIA for 58 (9.7%).

### 2.3. Measurements

#### 2.3.1. Sarcopenia

Sarcopenia, the dependent variable in this study, was defined according to the AWGS 2025 criteria as the concurrent presence of low muscle mass and low muscle strength [9]. Sarcopenia status was determined using handgrip strength and appendicular skeletal muscle mass measurements from KNHANES, with the corresponding variable names provided in parentheses below [19].

Muscle strength was assessed using handgrip strength measured with a digital grip strength dynamometer (T.K.K.5401, Niigata, Japan). The maximum valid value among the first and second measurements of the right hand (GS_mea_r_1, GS_mea_r_2) and left hand (GS_mea_l_1, GS_mea_l_2) was used. When measurements were available for only one hand, the maximum valid value for that hand was used [19].

For appendicular skeletal muscle mass, measurements obtained using dual-energy X-ray absorptiometry (DXA) were used preferentially. DXA-derived appendicular skeletal muscle mass was calculated by subtracting the bone mineral content of each limb (DW_Rrm_BMC, DW_Lrm_BMC, DW_Rlg_BMC, DW_Llg_BMC) from its lean mass including bone mineral content (DW_Rrm_LN, DW_Lrm_LN, DW_Rlg_LN, DW_Llg_LN) and summing the resulting values across both arms and legs. Values were converted from grams to kilograms. In KNHANES, DXA examinations may be restricted by exclusion criteria, including recent contrast imaging or nuclear medicine examinations, difficulty maintaining the required examination position, and body size exceeding the equipment’s scanning range [19]. However, the individual reasons for missing DXA measurements could not be determined in this study. When DXA measurements were unavailable but bioelectrical impedance analysis (BIA) measurements were available, BIA data were used. BIA-derived appendicular skeletal muscle mass was calculated as the sum of the muscle mass of both arms and legs, excluding bone mineral content (BIA_LRA, BIA_LLA, BIA_LRL, BIA_LLL). The appendicular skeletal muscle mass index (ASMI) was calculated by dividing appendicular skeletal muscle mass (kg) by the square of height (m^2^). Height was obtained from the HE_ht variable and converted from centimeters to meters.

The age- and measurement-specific AWGS 2025 criteria for Asian women were applied to determine sarcopenia status. Women aged 50–64 years were classified as having sarcopenia when they had both a handgrip strength of <20 kg and a DXA-derived ASMI of <5.5 kg/m^2^. Women aged ≥65 years were classified as having sarcopenia when they had both a handgrip strength of <18 kg and a DXA-derived ASMI of <5.4 kg/m^2^. For participants assessed using BIA, an ASMI cutoff of <5.7 kg/m^2^ was applied to define low muscle mass in both age groups, together with the corresponding age-specific handgrip strength cutoff [9]. Participants meeting both the low muscle mass and low muscle strength criteria were classified into the sarcopenia group, whereas all others were classified into the non-sarcopenia group.

#### 2.3.2. Blood Biochemical Parameters

Biochemical parameters were obtained from standardized blood tests performed as part of the KNHANES health examination [19]. The blood biochemical parameters were selected to represent multiple physiological domains, based on previous literature on metabolic, inflammatory, hematological, nutritional, and mineral-related factors associated with sarcopenia [1,12,13,14,15,16,17,18]. The variables included glycated hemoglobin (HbA1c; %), total cholesterol (mg/dL), high-density lipoprotein cholesterol (HDL-C; mg/dL), triglycerides (mg/dL), hemoglobin (g/dL), creatinine (mg/dL), aspartate aminotransferase (AST; U/L), high-sensitivity C-reactive protein (hs-CRP; mg/L), calcium (mg/dL), phosphorus (mg/dL), magnesium (mg/dL), parathyroid hormone (PTH; pg/mL), and serum 25-hydroxyvitamin D_3_ [25(OH)D_3_; ng/mL].

#### 2.3.3. General Characteristics

General characteristics were categorized as sociodemographic, physical and menopause-related, and health–behavioral characteristics. Sociodemographic characteristics included age, household income level, educational level, and economic activity status. These variables were analyzed according to the classifications provided in the KNHANES raw dataset.

Physical and menopause-related characteristics included body mass index (BMI), age at menopause, and duration of hormone replacement therapy (HRT). Age at menopause was defined as the self-reported age at natural or surgical menopause. BMI was calculated as body weight (kg) divided by the square of height (m^2^), using measurements obtained during the health examination. It was analyzed as a continuous variable in the regression analyses and categorized as underweight (<18.5 kg/m^2^), normal weight (18.5 ≤ BMI < 23.0 kg/m^2^), overweight (23.0 ≤ BMI < 25.0 kg/m^2^), or obesity (≥25.0 kg/m^2^) to describe participant characteristics. The duration of HRT was analyzed as a continuous variable in months. Participants who reported never having received hormone replacement therapy were assigned a duration of 0 months.

Health–behavioral characteristics included lifetime smoking, monthly alcohol consumption, and muscle-strengthening exercise frequency (days/week). Lifetime smoking was categorized as never having smoked or having smoked fewer than 100 cigarettes (five packs) during one’s lifetime versus having smoked at least 100 cigarettes. Monthly alcohol consumption was categorized as lifetime abstinence or drinking less than once per month during the previous year versus drinking at least once per month during the previous year. Muscle-strengthening exercise frequency (days/week) was measured as the number of days participants performed exercises such as push-ups or sit-ups during the previous week and was treated as a continuous variable ranging from 0 (none) to 7 days.

### 2.4. Statistical Analysis

Data were analyzed using IBM SPSS Statistics version 31.0 (IBM Corp., Armonk, NY, USA). Because KNHANES uses a two-stage stratified cluster sampling design, with enumeration districts and households as the primary and secondary sampling units, respectively, complex sample analyses were performed using the stratification variable (kstrata), cluster variable (PSU), and examination weight (wt_itvex). Data from all 6997 survey participants were retained, and the 1157 postmenopausal women who met the final eligibility criteria were designated as the analytical subpopulation to account for the full survey design.

Participants’ general characteristics and biochemical parameters were summarized using complex sample frequency and descriptive analyses. Categorical variables were presented as unweighted frequencies and weighted percentages, and continuous variables as weighted means and standard errors. Differences by sarcopenia status were assessed using Rao–Scott adjusted F tests in complex sample crosstabulation analyses for categorical variables and Wald F tests in complex sample general linear models for continuous variables. Descriptive analyses and between-group comparisons used the available observations for each variable.

Complex sample logistic regression analyses were performed to examine the associations between blood biochemical parameters and sarcopenia. Each biochemical parameter was examined in a separate regression model; all biochemical parameters were not entered simultaneously into a single model. Model 1 included the biochemical parameter of interest and age, whereas Model 2 included the biochemical parameter of interest, age, body mass index (BMI), and age at menopause. All regression models used the same 1157 participants with complete data for all model variables, including 121 sarcopenia cases. Covariates were selected based on previous research, considering age as a major factor associated with declines in muscle mass and strength, BMI as a factor related to body composition and muscle health, and age at menopause as an indicator of the timing of menopause-related hormonal changes potentially associated with muscle health [1,7,12,20].

Biochemical parameters were entered as continuous variables. Associations with sarcopenia were reported as adjusted odds ratios with 95% confidence intervals, Wald F statistics, and *p*-values for each one-unit increase in biochemical parameters; triglyceride associations were additionally expressed per 10 mg/dL increase. Variance inflation factors for age, BMI, and age at menopause ranged from 1.000 to 1.013, indicating no evidence of multicollinearity among these covariates. All statistical tests were two-sided, and the significance level before multiple-comparison adjustment was set at *p* < 0.05.

To account for multiple comparisons across biochemical parameters, false discovery rate (FDR) adjustment was performed using the Benjamini–Hochberg procedure. The *p*-values for the 13 biochemical parameters were adjusted separately within Model 1 and Model 2, with each model constituting a distinct family of tests. Both nominal *p*-values and FDR-adjusted *p*-values (q-values) were reported. Associations were interpreted considering effect sizes and 95% confidence intervals, with statistical significance after multiple-comparison adjustment defined as q < 0.05.

### 2.5. Ethical Considerations

The KNHANES 2024 protocol was approved by the Institutional Review Board of the Korea Disease Control and Prevention Agency (approval no. 2022-11-16-R-03), and written informed consent was obtained from all participants before their participation in the survey. The present study was a secondary analysis of publicly available KNHANES data. The researcher agreed to the requirements regarding personal information protection and compliance with the terms of data use before downloading the dataset. All personally identifiable information had been removed before the data were made available. Therefore, additional institutional review board approval was not required for the present study.

## 3. Results

### 3.1. Prevalence of Sarcopenia and Differences in General Characteristics According to Sarcopenia Status

The prevalence of sarcopenia and differences in general characteristics according to sarcopenia status are presented in Table 1. The study included 1157 postmenopausal women aged ≥50 years, representing an estimated population of 6,330,964 after the complex survey weights were applied. The overall weighted prevalence of sarcopenia was 10.5% (95% CI: 8.7–12.7%). By age group, the prevalence was 9.7% (95% CI: 7.4–12.6%) among women aged 50–64 years and 11.4% (95% CI: 8.7–14.9%) among women aged ≥65 years. The difference between the two age groups was not statistically significant (*F* = 0.781, *p* = 0.378).

Among the sociodemographic and health–behavioral characteristics, age and household income level differed significantly by sarcopenia status. The weighted mean age was significantly higher in the sarcopenia group than in the non-sarcopenia group (*F* = 5.310, *p* = 0.023). Compared with the non-sarcopenia group, the sarcopenia group had a higher proportion of women in the lowest household-income category and a lower proportion in the highest household-income category (*F* = 3.157, *p* = 0.027). No significant differences were observed in economic activity status (*F* = 3.735, *p* = 0.055), educational level (*F* = 2.250, *p* = 0.084), lifetime smoking (*F* = 0.025, *p* = 0.874), monthly alcohol consumption (*F* = 0.213, *p* = 0.645), or muscle-strengthening exercise (*F* = 1.921, *p* = 0.168).

Regarding physical and menopause-related characteristics, mean BMI was significantly lower in the sarcopenia group (*F* = 24.506, *p* < 0.001). The distribution of BMI categories also differed significantly according to sarcopenia status (*F* = 3.232, *p* = 0.028). The sarcopenia group had a higher proportion of women in the normal-weight category and a lower proportion in the obesity category than the non-sarcopenia group. The duration of HRT was significantly shorter in the sarcopenia group (*F* = 3.939, *p* = 0.049). Age at menopause did not differ significantly between the groups (*F* = 3.793, *p* = 0.053).

### 3.2. Differences in Biochemical Parameters According to Sarcopenia Status

Differences in blood biochemical parameters by sarcopenia status are presented in Table 2. Compared with the non-sarcopenia group, the sarcopenia group had significantly lower levels of hemoglobin (*F* = 9.793, *p* = 0.002), hs-CRP (*F* = 6.894, *p* = 0.010), and calcium (*F* = 5.504, *p* = 0.020). No significant differences were observed between the groups in HbA1c, total cholesterol, HDL-C, triglycerides, creatinine, AST, phosphorus, magnesium, PTH, or serum 25(OH)D_3_ (all *p* > 0.05).

### 3.3. Adjusted Associations Between Biochemical Parameters and Sarcopenia Status in Postmenopausal Women

The associations between blood biochemical parameters and sarcopenia in postmenopausal women in the age-adjusted model (Model 1) are presented in Table 3.

Higher hemoglobin (aOR = 0.734, 95% CI = 0.599–0.901, *p* = 0.003) and calcium levels (aOR = 0.559, 95% CI = 0.319–0.981, *p* = 0.043) were associated with lower odds of sarcopenia. After FDR correction, only hemoglobin remained statistically significant (*q* = 0.039), whereas calcium was no longer statistically significant (*q* = 0.280).

The associations between blood biochemical parameters and sarcopenia in the model adjusted for age, BMI, and age at menopause (Model 2) are presented in Table 4.

Higher high-density lipoprotein cholesterol (HDL-C; aOR = 0.985, 95% CI = 0.971–0.999, *p* = 0.031) and hemoglobin levels (aOR = 0.782, 95% CI = 0.629–0.972, *p* = 0.027) were associated with lower odds of sarcopenia. Conversely, higher triglyceride levels were associated with higher odds of sarcopenia (aOR = 1.005, 95% CI = 1.002–1.007, *p* < 0.001).

After FDR correction, only the association with triglycerides remained statistically significant (q < 0.013). The associations with HDL-C and hemoglobin were not supported after correction for multiple testing (both q = 0.134). The odds ratio for triglycerides represents the change in odds per 1 mg/dL increase. To facilitate interpretation of the effect size, rescaling this estimate to a 10 mg/dL increase corresponded to approximately 5.1% higher odds of sarcopenia (aOR = 1.051, 95% CI = 1.020–1.072). No other biochemical parameters were significantly associated with sarcopenia after FDR correction (all *q* > 0.05).

## 4. Discussion

This study applied the AWGS 2025 diagnostic criteria to estimate the prevalence of sarcopenia among postmenopausal women aged ≥50 years and to examine associations between blood biochemical parameters and sarcopenia in models adjusted for age and for age, BMI, and age at menopause. The overall weighted prevalence of sarcopenia was 10.5%, numerically similar to the prevalence of 9.7% among women reported by Kim et al. in a study of Korean adults aged ≥65 years [10]. An international systematic review and meta-analysis reported a wide range of sarcopenia prevalence estimates, from 10% to 27%, depending on the diagnostic criteria and cutoffs [5]. The pooled prevalence in studies applying the EWGSOP2 criteria was 10%, similar to the present findings [5]. However, direct comparisons require caution because of differences in participants’ age and sex distributions and diagnostic criteria. In particular, this study applied different handgrip strength and DXA-derived ASMI cutoffs to women aged 50–64 years and those aged ≥65 years according to the AWGS 2025 criteria. These age-specific differences in diagnostic thresholds should therefore also be considered when comparing prevalence estimates across studies [9].

International studies applying the EWGSOP2 criteria to postmenopausal women have also reported substantial variation in prevalence. Zanchetta et al. reported a sarcopenia prevalence of 4.0% among 250 postmenopausal women aged >60 years in Argentina [21], whereas Pap et al. reported a prevalence of 31.0% among 100 postmenopausal women aged 50–84 years in Hungary [22]. Although the prevalence of 10.5% in this study falls between these estimates, differences in sample characteristics, age distribution, health status, exclusion criteria, diagnostic criteria, and measurement methods make it difficult to interpret these findings as differences in sarcopenia burden across countries [9,21,22]. International comparisons of prevalence should therefore consider differences in participant selection and measurement methods alongside diagnostic criteria.

In this study, the prevalence of sarcopenia among postmenopausal women aged 50–64 years was 9.7%, numerically lower than the 11.4% among women aged ≥65 years, although the difference between the two groups was not statistically significant. Using the same dataset, Yoo et al. reported low muscle mass prevalence estimates of 3.2–3.4% across five-year age groups among all women aged 50–64 years, substantially different from the present findings [4]. Whereas Yoo et al. estimated prevalence based solely on DXA-derived low muscle mass, this study included only postmenopausal women aged 50–64 years, incorporated BIA measurements when DXA measurements were unavailable, and applied both muscle mass and handgrip strength criteria. Differences in classification criteria and measurement methods should therefore be considered when interpreting these estimates. The present findings suggest a need to extend preventive muscle health care, traditionally emphasized for adults aged ≥65 years, to individuals before older age, consistent with the intent of the AWGS 2025 consensus.

A notable finding of this study was that HDL-C and triglycerides were not significantly associated with sarcopenia in the age-adjusted model but were associated with lower and higher odds of sarcopenia, respectively, in the model adjusted for age, BMI, and age at menopause, before FDR adjustment. Both models used the same analytical sample; therefore, the changes in the estimated associations reflected covariate adjustment rather than differences in sample composition. However, only the association with triglycerides remained statistically significant after FDR adjustment; the association with HDL-C should therefore be interpreted cautiously as an exploratory finding.

BMI and age at menopause may help explain the observed changes in the lipid association estimates. In this study, BMI was significantly lower in the sarcopenia group than in the non-sarcopenia group, consistent with a previous study identifying BMI as a major factor in classifying sarcopenia risk groups among older Korean women [10]. Because BMI is related to body composition and lipid metabolism, adjusting for it accounts for differences in body size when estimating associations between lipid parameters and sarcopenia. However, BMI does not distinguish muscle mass from fat mass. Greendale et al. reported that fat mass increased while lean mass decreased during the menopausal transition, indicating that body weight alone may not adequately capture changes in body composition [6]. Accordingly, the between-group difference in BMI observed in this study should not be interpreted as evidence of a protective effect of higher BMI or obesity, nor should BMI adjustment alone be considered sufficient to control for the influence of body composition.

Although age at menopause did not differ significantly by sarcopenia status in this study, this between-group comparison alone does not indicate that it need not be considered in association analyses. Jeong et al. reported that early menopause at age ≤45 years was associated with lower total lean body mass and, in certain BMI groups, higher odds of sarcopenia [20]. A systematic review and meta-analysis by Divaris et al. also reported associations of early menopause with lower muscle mass and premature ovarian insufficiency with lower handgrip strength [8]. Together with previous literature addressing the biological relationship between estrogen and muscle health [7], these findings support accounting for differences in menopausal timing when evaluating associations between lipid parameters and sarcopenia. However, direct comparisons are limited because previous studies assessed early menopause categorically, whereas age at menopause was entered as a continuous variable in this study. Furthermore, because BMI and age at menopause were added simultaneously, their individual contributions to the changes in the lipid association estimates could not be distinguished, limiting interpretation.

In the model adjusted for age, BMI, and age at menopause, each 10 mg/dL increase in triglyceride levels was associated with approximately 5.1% higher odds of sarcopenia. This association suggests the need to further examine the relationship between lipid metabolism and sarcopenia among postmenopausal women. Previous Korean studies have reported triglycerides as a factor associated with sarcopenia in adults [17] and have included HDL-C and triglycerides among the factors used to classify sarcopenia risk groups in older women [10]. Using data from the US National Health and Nutrition Examination Survey, Huang et al. reported that a higher triglyceride-to-HDL-C ratio was associated with lower BMI-adjusted relative grip strength in adults aged ≥60 years, with a similar association observed in the female subgroup [23]. Although direct comparisons with the present study are limited because that study examined the ratio of the two lipid parameters and relative grip strength, its findings provide supporting evidence for an association between lipid metabolism and muscle function.

Dysregulated lipid metabolism and intramuscular fat accumulation may contribute to reductions in muscle mass and strength through inflammation, oxidative stress, insulin resistance, and mitochondrial dysfunction [12]. In postmenopausal women, increases and redistribution of body fat associated with declining estrogen levels may also contribute to these interactions [24]. However, these mechanisms were not directly evaluated in this study, and potential confounders, including lipid-lowering medication use, physical activity, diet, and comorbidities, were not adjusted for. Hormone replacement therapy duration was also significantly shorter in the sarcopenia group but was not included as an adjustment covariate, leaving the possibility of residual confounding related to treatment history. Therefore, the association with triglycerides that remained significant after FDR adjustment should be interpreted as an association observed after adjustment for age, BMI, and age at menopause; it does not establish independence from other relevant factors or a causal relationship. The association with HDL-C, which did not remain significant after correction for multiple testing, should be interpreted cautiously as an exploratory finding and requires confirmation in studies accounting for additional confounders, body composition, and hormone replacement therapy history.

Hemoglobin levels were significantly lower in the sarcopenia group than in the non-sarcopenia group. In the age-adjusted model, higher hemoglobin levels were associated with lower odds of sarcopenia, and this association remained statistically significant after FDR adjustment. In the model additionally adjusted for BMI and age at menopause, the association remained significant before FDR adjustment but was no longer statistically significant afterward. These findings indicate that the statistical evidence for the hemoglobin association varied with model specification and multiple-testing adjustment, suggesting that BMI and age at menopause warrant careful consideration when interpreting this association.

The finding of lower hemoglobin levels in the sarcopenia group is consistent with a systematic review and meta-analysis reporting significantly lower hemoglobin levels in individuals with sarcopenia in both the overall analysis and the subgroup analysis of studies using AWGS criteria [13]. Tseng et al. also reported that anemia was associated with sarcopenia among older adults and that higher hemoglobin levels were associated with greater handgrip strength [14]. Given the oxygen-carrying function of hemoglobin, an association between lower hemoglobin levels and impaired muscle function is biologically plausible. Further studies are needed to assess the reproducibility of this association and its potential clinical relevance.

Serum calcium levels were significantly lower in the sarcopenia group, and higher calcium levels were associated with lower odds of sarcopenia in the age-adjusted model. However, this association was not statistically significant after FDR correction, and no significant association was observed in the model adjusted for age, BMI, and age at menopause. Therefore, the association between calcium and sarcopenia should be interpreted cautiously as an exploratory finding. Serum inorganic phosphorus, magnesium, and 25-hydroxyvitamin D_3_ also showed no significant associations with sarcopenia. A systematic review of older adults likewise found no significant association between calcium and sarcopenia-related outcomes, whereas findings for phosphorus were inconsistent [16]. In contrast, moderate evidence supported significant associations of magnesium with muscle mass, muscle strength, physical performance, and sarcopenia prevalence, which differs from the present findings [16].

The present findings regarding vitamin D differed from those of Bae and Kim, who reported a significant association between blood vitamin D concentrations and sarcopenia among Korean adults [17], and from those of Luo et al., whose meta-analysis reported lower blood 25-hydroxyvitamin D [25(OH)D] concentrations in individuals with sarcopenia than in those without sarcopenia [18]. However, Luo et al. pooled between-group differences in blood concentrations, whereas the present study examined associations among postmenopausal women after adjustment for age, BMI, and age at menopause; therefore, direct comparisons should be made cautiously. Serum 25(OH)D_3_ measured in the present study should also be distinguished from blood 25(OH)D evaluated in previous studies. Differences in participant characteristics, sarcopenia diagnostic criteria, muscle-mass assessment methods, baseline nutritional status, vitamin D measures, and covariate adjustment may partly explain the differences in findings.

Meanwhile, the meta-analysis by Prokopidis et al. found that vitamin D supplementation alone did not significantly improve handgrip strength, appendicular lean mass, or overall muscle strength [25]. This highlights the need to distinguish observational associations between blood vitamin D concentrations and sarcopenia from the effects of vitamin D supplementation interventions. Furthermore, serum mineral and vitamin D metabolite concentrations are influenced by dietary intake, renal function, hormonal regulation, and physiological homeostasis; therefore, a single measurement may not adequately reflect long-term status or intracellular availability. Longitudinal and intervention studies are needed to clarify the associations between these indicators and sarcopenia and the effects of vitamin D supplementation among postmenopausal women.

A strength of this study is its use of nationally representative data and complex survey weights, which enhanced the generalizability of the findings to Korean postmenopausal women aged ≥50 years. In addition to self-reported characteristics, the study used objective examination data collected through standardized blood tests, handgrip strength measurements, and assessments of body composition and appendicular skeletal muscle mass using DXA or BIA. Furthermore, the newly revised AWGS 2025 diagnostic criteria were applied, allowing the inclusion of postmenopausal women aged 50–64 years rather than limiting the study population to older adults. The study thus contributes by characterizing sarcopenia prevalence from midlife through older age and exploring its associations with menopause-related characteristics and a range of biochemical indicators measured in the same participants.

Several limitations should also be considered. First, the cross-sectional design precludes determination of temporal or causal relationships between sarcopenia and the associated factors. Second, biochemical indicators based on blood test results reflect physiological status at a single point in time and may not adequately represent long-term changes. Third, although the analyses adjusted for age, BMI, and age at menopause based on previous research, residual confounding may remain because socioeconomic and lifestyle factors, detailed dietary intake, comorbidities, medication use, and other potentially relevant factors were not fully accounted for. Fourth, DXA measurements were used preferentially to classify sarcopenia, but BIA measurements were used for 87 participants (7.5%) without DXA measurements. Although measurement-specific thresholds from the AWGS 2025 consensus were applied, differences between the two methods may have affected sarcopenia classification and the estimated associations. Fifth, excluding participants with missing handgrip strength, appendicular skeletal muscle mass index, or blood biochemical parameters may have introduced selection bias. Functional limitations or health conditions affecting the ability to undergo testing may have resulted in differences in sarcopenia risk between included and excluded participants, potentially influencing the prevalence estimate and the observed associations. Sixth, menopausal status was classified based on self-reported responses, and ovarian conservation at hysterectomy could not be determined. Despite the inclusion criterion of age ≥50 years, some women who underwent hysterectomy with ovarian conservation may not have reached physiological menopause, potentially resulting in misclassification of menopausal status. Finally, the findings were obtained from Korean postmenopausal women and may not be directly generalizable to women from other racial, ethnic, or healthcare contexts.

Future longitudinal studies are needed to clarify the temporal relationships between changes in body composition, lipid metabolism, hematological status, and sarcopenia after menopause. Studies incorporating repeated biochemical measurements and comprehensive information on dietary intake, physical activity, comorbidities, medication use, and menopause-related characteristics are also needed. Before these parameters can be used to identify women for early muscle health assessment and prevention, further research is required to establish the reproducibility of the findings across diverse populations and validate their clinical utility.

## 5. Conclusions

This study applied the AWGS 2025 diagnostic criteria to estimate the prevalence of sarcopenia among Korean postmenopausal women aged ≥50 years and explore its associations with menopause-related characteristics and biochemical parameters. The weighted prevalence of sarcopenia was 10.5% overall and 9.7% among women aged 50–64 years, suggesting the need for attention to muscle health and preventive care before older age. Triglyceride levels were not significantly associated with sarcopenia in the age-adjusted model; however, in the model adjusted for age, body mass index, and age at menopause, each 10 mg/dL higher triglyceride level was associated with approximately 5.1% higher odds of sarcopenia. Higher hemoglobin levels were also associated with lower odds of sarcopenia in the age-adjusted model, but the association was not statistically significant after accounting for multiple testing in the model adjusted for age, body mass index, and age at menopause. This study provides preliminary evidence for exploring the potential use of blood biochemical parameters in muscle health assessment and management among postmenopausal women, although further validation is needed before clinical application.

## Figures and Tables

**Table 1:** Differences in general characteristics according to sarcopenia status.

Characteristics	Categories	*n* (W%) * or Mean ± SE **		Rao–Scott or Wald *F*	*p*
		Non-Sarcopenia 1036 (89.5)	Sarcopenia 121 (10.5)		
Age (years)		64.38 ± 0.33	66.78 ± 1.05	5.31	0.023
Age group (years)	50–64	496 (90.3)	52 (9.7)	0.781	0.378
	≥65	540 (88.6)	69 (11.4)		
Economic activity	Yes	532 (52.9)	50 (42.5)	3.735	0.055
	No	504 (47.1)	71 (57.5)		
Income level	Low	259 (22.7)	47 (34.4)	3.157	0.027
	Lower-middle	278 (26.3)	29 (28.6)		
	Upper-middle	235 (22.8)	26 (20.4)		
	High	264 (28.3)	19 (16.6)		
Education level	≤Elementary school	316 (27.2)	53 (39.0)	2.25	0.084
	Middle school	178 (16.9)	19 (16.8)		
	High school	335 (33.2)	33 (28.9)		
	≥College	207 (22.7)	16 (15.3)		
Lifetime smoking	No	981 (94.8)	113 (94.5)	0.025	0.874
	Yes	55 (5.2)	8 (5.5)		
Alcohol consumption *** (time/month)	<1	722 (68.5)	81 (66.1)	0.213	0.645
	≥1	311 (31.5)	38 (33.9)		
Muscle-strengthening exercise (days/week)		0.80 ± 0.05	0.57 ± 0.15	1.921	0.168
BMI (kg/m^2^)		23.89 ± 0.12	22.45 ± 0.26	24.506	<0.001
BMI category (kg/m^2^)	≥25.0	357 (33.6)	24 (21.3)	3.232	0.028
	23.0–24.9	242 (23.6)	21 (19.3)		
	18.5–22.9	406 (39.7)	67 (53.0)		
	<18.5	31 (3.2)	9 (6.5)		
Age at menopause (years)		50.62 ± 0.13	49.86 ± 0.37	3.793	0.053
HRT duration (months)		6.50 ± 0.74	3.29 ± 1.44	3.939	0.049

* *n* = unweighted sample size; W% = weighted percentage. Percentages for categorical variables are calculated within each sarcopenia-status group, except for age groups, for which percentages are calculated within each age group. ** SE = standard error. *** alcohol consumption: five missing responses (non-sarcopenia, *n* = 3; sarcopenia, *n* = 2); available-case analysis. The total sample comprised 1157 participants, representing a weighted population of 6,330,964.

**Table 2:** Differences in blood biochemical parameters according to sarcopenia status.

Biochemical Parameters	Mean ± SE *		Wald *F*	*p*
	Non-Sarcopenia (*n* = 1036)	Sarcopenia (*n* = 121)		
HbA1c (%)	5.79 ± 0.02	5.82 ± 0.07	0.105	0.747
Total cholesterol (mg/dL)	187.85 ± 1.66	186.29 ± 4.40	0.120	0.730
HDL-C (mg/dL)	61.84 ± 0.51	60.33 ± 1.41	0.893	0.346
Triglycerides (mg/dL)	114.18 ± 1.97	127.99 ± 8.66	2.313	0.130
Hemoglobin (g/dL)	13.18 ± 0.03	12.83 ± 0.11	9.793	0.002
Creatinine (mg/dL)	0.712 ± 0.005	0.712 ± 0.019	<0.001	0.984
AST (SGOT, U/L)	24.52 ± 0.56	23.29 ± 0.61	2.375	0.125
hs-CRP (mg/L)	1.14 ± 0.12	0.75 ± 0.09	6.894	0.010
Calcium (mg/dL)	9.56 ± 0.02	9.47 ± 0.03	5.504	0.020
Phosphorus (mg/dL)	3.83 ± 0.02	3.80 ± 0.04	0.557	0.457
Magnesium (mg/dL)	2.144 ± 0.005	2.126 ± 0.019	0.889	0.347
PTH (pg/mL)	38.13 ± 0.63	36.82 ± 1.33	0.813	0.369
Serum 25(OH)D_3_ (ng/mL)	30.48 ± 0.43	31.27 ± 1.40	0.298	0.586

* SE = standard error. Total unweighted sample size: 1157; weighted population estimate: 6,330,964.

**Table 3:** Age-adjusted associations between biochemical parameters and sarcopenia status in postmenopausal women.

Biochemical Parameters	aOR (95% CI) *	Wald *F*	*p*	FDR (*q*) **
HbA1c (%)	0.974 (0.698–1.357)	0.025	0.874	0.874
Total cholesterol (mg/dL)	1.001 (0.996–1.006)	0.110	0.741	0.874
HDL-C (mg/dL)	0.997 (0.984–1.009)	0.289	0.591	0.874
Triglycerides (mg/dL)	1.003 (1.000–1.006)	3.122	0.079	0.342
Hemoglobin (g/dL)	0.734 (0.599–0.901)	8.889	0.003	0.039
Creatinine (mg/dL)	0.708 (0.098–5.098)	0.119	0.730	0.874
AST (SGOT, U/L)	1.014 (0.992–1.036)	1.626	0.204	0.530
hs-CRP (mg/L)	0.906 (0.799–1.028)	2.379	0.125	0.406
Calcium (mg/dL)	0.559 (0.319–0.981)	4.172	0.043	0.280
Phosphorus (mg/dL)	0.957 (0.621–1.476)	0.039	0.843	0.874
Magnesium (mg/dL)	0.472 (0.095–2.338)	0.860	0.355	0.659
PTH (pg/mL)	1.008 (0.994–1.023)	1.280	0.260	0.563
Serum 25(OH)D_3_ (ng/mL)	1.003 (0.986–1.021)	0.143	0.706	0.874

* aOR = adjusted odds ratio; CI = confidence interval. ** FDR = false discovery rate; q = Benjamini–Hochberg FDR-adjusted *p*-value. Each biochemical parameter was analyzed in a separate complex sample logistic regression model adjusted for age. All models included 1157 participants, representing a weighted population of 6,330,964.

**Table 4:** Associations between biochemical parameters and sarcopenia status in postmenopausal women adjusted for age, BMI, and age at menopause.

Biochemical Parameters	aOR (95% CI) *	Wald *F*	*p*	FDR (*q*) **
HbA1c (%)	1.142 (0.868–1.503)	0.917	0.340	0.552
Total cholesterol (mg/dL)	0.999 (0.994–1.004)	0.113	0.737	0.871
HDL-C (mg/dL)	0.985 (0.971–0.999)	4.762	0.031	0.134
Triglycerides (mg/dL)	1.005 (1.002–1.007)	11.480	<0.001	<0.013
Hemoglobin (g/dL)	0.782 (0.629–0.972)	4.988	0.027	0.134
Creatinine (mg/dL)	0.846 (0.121–5.901)	0.029	0.865	0.882
AST (SGOT, U/L)	1.015 (0.994–1.037)	1.898	0.170	0.368
hs-CRP (mg/L)	0.940 (0.875–1.009)	2.947	0.088	0.229
Calcium (mg/dL)	0.578 (0.315–1.059)	3.203	0.076	0.229
Phosphorus (mg/dL)	0.849 (0.545–1.323)	0.533	0.466	0.606
Magnesium (mg/dL)	0.373 (0.076–1.832)	1.498	0.223	0.414
PTH (pg/mL)	1.005 (0.991–1.020)	0.550	0.460	0.606
Serum 25(OH)D_3_ (ng/mL)	0.999 (0.982–1.016)	0.022	0.882	0.882

* aOR = adjusted odds ratio; CI = confidence interval. ** FDR = false discovery rate; q = Benjamini–Hochberg FDR-adjusted *p*-value. Each biochemical parameter was analyzed in a separate complex sample logistic regression model adjusted for age, BMI, and age at menopause. All models included 1157 participants, representing a weighted population of 6,330,964.

## Data Availability

The data presented in this study are openly available in the Korea National Health and Nutrition Examination Survey (KNHANES) at https://knhanes.kdca.go.kr/knhanes/eng/main.do (accessed on 15 September 2026).

## Abbreviations

The following abbreviations are used in this manuscript:

AST	Aspartate aminotransferase
AWGS	Asian Working Group for Sarcopenia
ASMI	Appendicular skeletal muscle mass index
BIA	Bioelectrical impedance analysis
BMI	Body mass index
DXA	Dual-energy X-ray absorptiometry
HbA1c	Glycated hemoglobin
HDL-C	High-density lipoprotein cholesterol
HRT	Hormone replacement therapy
hs-CRP	High-sensitivity C-reactive protein
KNHANES	Korea National Health and Nutrition Examination Survey
PTH	Parathyroid hormone
Serum 25(OH)D_3_	Serum 25-Hydroxyvitamin D_3_
